# Determinants of women’s satisfaction with maternal health care: a review of literature from developing countries

**DOI:** 10.1186/s12884-015-0525-0

**Published:** 2015-04-18

**Authors:** Aradhana Srivastava, Bilal I Avan, Preety Rajbangshi, Sanghita Bhattacharyya

**Affiliations:** Public Health Foundation of India, Plot no. 47, Sector 44, Institutional Area, Gurgaon, Haryana 122002 India; Faculty of infectious and tropical diseases, London School of Hygiene and Tropical Medicine, Keppel Street, London, WC1E 7HT UK

**Keywords:** Maternal satisfaction, Determinants, Quality of care, Deliveries, Developing countries

## Abstract

**Background:**

Developing countries account for 99 percent of maternal deaths annually. While increasing service availability and maintaining acceptable quality standards, it is important to assess maternal satisfaction with care in order to make it more responsive and culturally acceptable, ultimately leading to enhanced utilization and improved outcomes. At a time when global efforts to reduce maternal mortality have been stepped up, maternal satisfaction and its determinants also need to be addressed by developing country governments. This review seeks to identify determinants of women’s satisfaction with maternity care in developing countries.

**Methods:**

The review followed the methodology of systematic reviews. Public health and social science databases were searched. English articles covering antenatal, intrapartum or postpartum care, for either home or institutional deliveries, reporting maternal satisfaction from developing countries (World Bank list) were included, with no year limit. Out of 154 shortlisted abstracts, 54 were included and 100 excluded. Studies were extracted onto structured formats and analyzed using the narrative synthesis approach.

**Results:**

Determinants of maternal satisfaction covered all dimensions of care across structure, process and outcome. Structural elements included good physical environment, cleanliness, and availability of adequate human resources, medicines and supplies. Process determinants included interpersonal behavior, privacy, promptness, cognitive care, perceived provider competency and emotional support. Outcome related determinants were health status of the mother and newborn. Access, cost, socio-economic status and reproductive history also influenced perceived maternal satisfaction.

Process of care dominated the determinants of maternal satisfaction in developing countries. Interpersonal behavior was the most widely reported determinant, with the largest body of evidence generated around provider behavior in terms of courtesy and non-abuse. Other aspects of interpersonal behavior included therapeutic communication, staff confidence and competence and encouragement to laboring women.

**Conclusions:**

Quality improvement efforts in developing countries could focus on strengthening the process of care. Special attention is needed to improve interpersonal behavior, as evidence from the review points to the importance women attach to being treated respectfully, irrespective of socio-cultural or economic context. Further research on maternal satisfaction is required on home deliveries and relative strength of various determinants in influencing maternal satisfaction.

**Electronic supplementary material:**

The online version of this article (doi:10.1186/s12884-015-0525-0) contains supplementary material, which is available to authorized users.

## Background

Every year about 287,000 women die of causes associated with childbirth, 99 percent in developing countries [1]. Owing to considerable gaps in services, developing countries emphasize on increasing service availability and maintaining acceptable quality standards [2]. Understanding maternal perception of care and satisfaction with services is important in this regard, as perceived quality is a key determinant of service utilization [3,4]. Users who perceive the quality of care in a health center to be good, are more likely to visit it again, thereby increasing demand for the service [5,6]. Service utilization and positive maternal and neonatal outcomes can be significantly enhanced by improving quality of facility deliveries and making them more acceptable to women [7]. User satisfaction is considered ‘patient’s judgment on the quality and goodness of care’ [8]. Patient satisfaction is thus indispensible to quality improvement with regard to design and management of health care systems [4].

Maternal satisfaction has often been defined using theoretical models of patient satisfaction [9]. But there is consensus that it is a multidimensional concept, influenced by a variety of factors [9,10]. It is therefore also defined as “positive evaluation of distinct dimensions of childbirth” [11].

At a time when global efforts to reduce maternal mortality have been stepped up, it is important to look at maternal satisfaction and its determinants [12]. Evidence on women’s perception of and satisfaction with the quality of maternal care help determine other aspects of care that need strengthening in developing country contexts to support long-term demand, generate significant changes in maternal care-seeking behavior, and identify barriers that can and should be removed.

This review looks at the evidence on determinants of maternal satisfaction in developing countries. It answers the question - what are the various determinants of maternal satisfaction that emerge from the literature in the context of both home and institutional deliveries in developing countries? Determinants have been extracted and organized thematically on the basis of the classical Donabedian framework of dimensions of care, while the Hulton framework on quality of maternity care has been utilized to further classify the determinants across sub-themes [8,13].

## Methods

### Research approach

**Figure 1:**
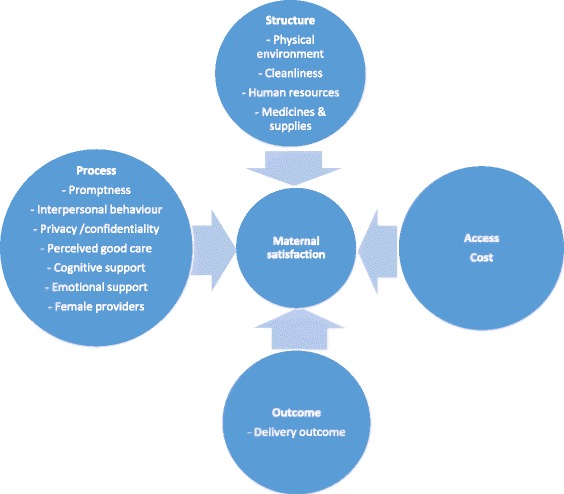
Conceptual framework of maternal satisfaction.

The classical Donabedian framework that categorizes dimensions of care into structure, process and outcome, has been used in many studies assessing patient perception [4,14]. Hulton et al. developed a framework specifically for quality of maternity care to facilitate assessment within institutional contexts [13]. This review follows the Donabedian framework in arranging major themes of determinants of satisfaction across the three dimensions of structure, process and outcome. Within the broad themes, sub-themes have been placed in the continuum of care approach, sequencing from antenatal and intra-partum to postnatal periods. The Hulton framework has also been utilized in defining themes and sub-themes based on the experience of care. Other elements not specific to quality frameworks, such as convenience of access, socio-economic and cultural determinants and maternal characteristics have also been included according to the evidence available around them (Figure 1).

### Review methodology

The review followed the methodology of systematic reviews. Review methodology was based on Cochrane collaboration as well as the NHS Centre for Reviews and Dissemination guidelines [15]. The review was part of the background research of a larger study. It follows the PRISMA framework. Both qualitative and quantitative studies were included in the review. The outcomes of interest were the determinants of maternal satisfaction as reported by the women covered in the studies. No scores or values were considered as the purpose was to identify the determinants and not the strength of their association with maternal satisfaction. No summary measure or meta-analysis was conducted.

### Search strategy

Key electronic databases of public health and social sciences were searched including PUBMED/MEDLINE, EMBASE, CINAHL, SCOPUS, POPLINE, PsycINFO, Sociological Abstracts, British Library Catalogue, CENTRAL, AEGIS, WHO Global Health Library, CAB abstracts, NLM Gateway (Clinical Trials Database), IndMED, WHO International Clinical Trials Registry and Google Scholar. Additionally, reference lists of relevant identified reviews and primary studies were searched. Grey literature was also sought through electronic searches as well as communication with experts. Table 1 lists the keywords used in the searches. All searches were conducted between January – March 2012 and updated between Oct-Dec 2013.

**Table 1:** **Keywords used in literature search (in various combinations for different databases)**

**Keywords**	**Associated search terms**
**1** ***. Maternity status:***	maternal OR mother OR woman OR pregnant OR “Pregnancy”[Mesh] OR “Maternal Health Services”[Mesh] AND (antenatal OR prenatal OR intrapartum OR childbirth OR delivery OR birthing OR postnatal OR postpartum)
**2** ***. Perception***:	perception OR opinion OR view OR knowledge
**3.** ***Maternal Satisfaction***:	satisfaction OR dignity OR autonomy OR confidentiality OR prompt attention OR care OR support OR amenities AND experience OR assessment
**4.** ***Location***:	“developing country” OR “developing countries” OR “middle income” OR “low income” OR “third world” OR poverty OR “resource poor” OR “poor country” OR “poor countries” OR “Developing Countries”[Mesh] OR “Poverty” OR “India”[Mesh]

### Eligibility criteria

#### Participants

The participants for the review included women from developing countries (low and middle income countries as classified by the World Bank) who have received maternal care services, including antenatal, intrapartum and postpartum care, and who reported on their satisfaction with the process. This could be a rating score or simply answering the question ‘are you satisfied with the care you received?’ Some studies from economically more developed countries like Taiwan, Argentina, Cuba and Saudi Arabia were also included as their findings were relevant for the review, especially on account of contextual similarities with emerging economies like China and India.

#### Interventions

No restriction was imposed by type of intervention. Both community and facility-based settings were included across all levels of care.

#### Comparison

Since there was no restriction by intervention design, there was also no restriction on the nature of comparisons.

#### Outcomes

Outcomes of interest were (i) any assessment of maternal satisfaction and (ii) determinants of maternal satisfaction.

### Exclusion criteria

Studies from developed countries, natural disaster, armed conflict and refugee contexts and studies in languages other than English were excluded. Articles discussing maternal satisfaction but not based on self-reported satisfaction were also excluded. Studies pertaining to only pain management or anesthesia during delivery were also excluded unless they reported on women’s satisfaction.

### Study selection

Study selection followed a two-stage process. In the first stage, abstracts of identified studies were screened by one researcher for relevance to the topic, based on the inclusion and exclusion criteria. In the second stage, full-text papers were reviewed by the same researcher for relevance and inclusion in the review. In case of any doubt, the researcher referred the concerned abstracts/full texts to two other researchers in the team and decided on inclusion based on their inputs.

### Data extraction

All findings relating to determinants of maternal satisfaction were included. Data was extracted electronically using a structured form by one researcher and checked for accuracy and detail by a second researcher (see Additional file 1).

Methodological quality was assessed on the basis of clarity of focus (clearly stated objective or research question), methodological rigor (scientific sampling method; validated tools; minimized risks) and robustness of evidence (clearly stated findings with confidence interval and levels of significance). In qualitative studies methodological rigor implied explanation of tool adaptation and internal consistency checks, and robustness of evidence implied clearly stated findings. Quality appraisal tool was based on the Strobe list, suitably modified for our review. It contained a list of 23 items on which studies were scored 1 or 0, depending on whether they fulfilled the criterion. Studies were graded as high, medium or low quality as per their scoring (less than 10 – low; 10-15 – moderate; more than 15 – high). However, in order to capture the range of determinants of maternal satisfaction, we did not use methodological quality as a criterion for inclusion in the review.

### Synthesis of results

The analysis followed a narrative synthesis approach, which is a textual approach to systematic review and synthesis of findings from multiple studies, telling the ‘story’ of the findings from the included studies [16]. The synthesis was developed manually, based on the extracted data.

### Risk of bias

We identified some potential risks of bias in the included studies, but did not assess them as the narrative synthesis did not require identification of risk levels. Facility based studies could suffer from the risk of inhibiting criticism of medical care [17]. Some studies list safeguards against these biases such as not involving health service personnel as interviewers [18,19], identifying participants from facility lists but interviewing outside the facility in neutral areas [17,20-24], or ensuring privacy in the place where interviews were conducted within the facility [25-32]. Women visiting the facility for the first time were also excluded from samples on the assumption of insufficient knowledge of quality related issues at the facility [25,29].

Other risks included convenience or limited sampling, which could lead to selection bias; non-representative sample and weak adaptation of instrument leading to social desirability bias.

Another possible bias with respect to client perception studies is the tendency to report satisfaction with services, influenced by positive outcomes (such as healthy mother and newborn after delivery). None of the studies, except one, explained or accounted for this potential bias.

### Ethical approval

Ethical approval for this study was granted by the Institutional Ethics Committee of the Public Health Foundation of India (TRC-IEC-187/13).

## Results

### Search outcome

**Figure 2:**
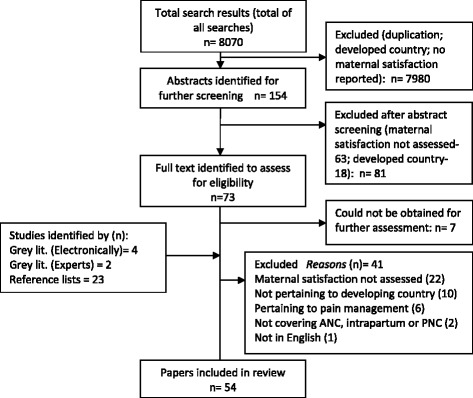
Flow Diagram summarizing searches.

The search results altogether yielded 8070 titles. After removal of duplication and irrelevant titles, 154 were identified for first-stage abstract screening. After screening these abstracts, 73 were identified for full-text retrieval. Out of these, seven texts could not be accessed. The remaining 66 were accessed and screened. Besides these, 29 other papers were identified from reference lists or manual grey literature searches. These were also included in the pool to yield a total of 95 full text articles that were retrieved and reviewed. After the final screening of full text articles, 54 studies were selected for inclusion in the review, while 41 were excluded as they either did not assess maternal satisfaction, pertain to developing country, cover ante-partum, intra-partum or post-partum care, were not in English or focused only on pain management (see Figure 2).

### Characteristics of studies selected for the review

All 54 selected studies, except two, were dated post 2000, indicating relatively recent interest in assessing maternal satisfaction in developing countries. All selected studies except one analyzed primary data. 26 studies were placed in Asia, 22 in Africa, three in Latin America and three were based on multi-country trials. Forty two were quantitative, six qualitative and six mixed-method studies. Facility-based interviews were conducted in 29 studies, while in 22 studies the interviews were based in the community. In three studies a combination of facility and community based interviews were conducted. Twenty studies focused on childbirth, 19 on antenatal care, four on postnatal care and 11 on maternal care in a broader manner. Most studies included in the review were of moderate quality (33 studies). Thirteen studies were of high quality while eight studies were of low quality. Some common drawbacks included insufficient justification or definition of independent variables (12 studies) and inadequate description of methods of analysis (22 studies). Measures of variability or confidence intervals were not provided in 16 studies. The included studies are summarized in Additional file 2: Table S2.

### Measurement of maternal satisfaction

Most of the studies used questionnaires to capture information on maternal satisfaction. Queries ranged from binary ‘yes/no’ type of questions to multiple-item scales for scoring levels of maternal satisfaction. In 26 studies questionnaires exclusively focused on capturing women’s perception and satisfaction with care. In 20 studies satisfaction was assessed through specific sections or queries on maternal satisfaction within a broader questionnaire. This was largely the case when maternal satisfaction was not the primary outcome variable. Among the qualitative studies, instruments included in-depth interview schedules and focus-group discussion guides (in whole or part) designed to capture information on maternal satisfaction. Among tools to assess levels of satisfaction, Likert-type rating scales were used in 19 studies, while the Visual Analog scale was used in four studies. The range of the scales varied from four to 20 points depending on the number of variables considered for evaluating satisfaction.

### Women’s overall satisfaction with maternal care

Most studies reported maternal satisfaction in terms of the proportion of women expressing satisfaction with maternal care. Ratings of maternal satisfaction indicate generally high ratings across developing countries. In 24 studies, more than 75 percent of the women reported care to be satisfactory. In 10 studies the proportion ranged between 50-75 percent, while in only three studies, it was less than 50 percent. Nine studies discussed ratings in terms of mean scores. Eight studies did not report any specific numerical value of satisfaction as they were qualitative in nature. They reported women’s expression of satisfaction or dissatisfaction with their experience of care.

### Determinants of maternal satisfaction

A large spectrum of determinants influencing maternal satisfaction emerged from this review. They are summarized here according to the Donabedian framework of structure, process and outcome, besides access, socioeconomic determinants and other determinants.

### Structure

#### Physical environment

Good physical environment and efficient management were significant in women’s positive assessment of the health facility and maternal care services [20,33-35]. These included good building infrastructure with water supply, electricity, beds, cleanliness, adequate room space, seating arrangement and waiting areas, as found in India, Bangladesh and Nigeria [27,36-38]. In Bangladesh, mothers who rated the availability of services at the facility (a composite of waiting area, drinking water, clean toilet and waiting time) as ‘good’ were significantly more satisfied with care than those who rated the services as ‘poor’ [27]. Efficient management improved patient’s access to services and streamlined patient consultations [27].

#### Cleanliness

Cleanliness, good housekeeping services and maintenance of hygiene were reported as a determinants of satisfaction in studies in Bangladesh, Gambia, Thailand, India and Iran [22,27,34,36,39,40]. Good housekeeping service emerged as a significant predictor of satisfaction with nursing care in a facility-based study in Thailand [39]. Frequent changing of bed sheets led to enhanced satisfaction with care in Gambia [22].

#### Availability and adequacy of human resources

Availability of doctors and nurses, especially during emergencies, was considered a prerequisite for good care in India and China [36,41]. Availability of doctors and nurses at all times, especially during emergencies, was considered a prerequisite to good care in India [36]. Non-availability of nursing personnel and inadequacy of staff to attend to women, especially during labor, was reported as a cause for dissatisfaction with services in Ghana and Nigeria [25,33].

#### Availability of medicines, supplies and services

Availability of prescription drugs, essential equipment like blood pressure monitors or thermometers, lab services and emergency supplies like blood and transfusion services, were reported as significant predictors of satisfaction with care in studies in India, Oman, Nigeria, Gambia and Uganda [22,25,26,35,36,42-44]. Occasional non-availability of essential medicines emerged as a cause for dissatisfaction with services in India and Nigeria [25,42]. Difficulties in arranging for supplies during emergencies, such as blood supply and transfusion services, were associated with dissatisfaction with care in Gambia [22].

### Process

#### Promptness of care

Prompt attention is a significant determinant of maternal satisfaction [19,45]. Prolonged waiting time is an important determinant of satisfaction with services and also figures prominently in women’s recollections of deficiency in services in Argentina, Gambia, Iran, Malawi, Nigeria, Sri Lanka, Saudi Arabia and Uganda [19,30,32,34,35,44,46-49]. Reducing waiting time was an important aspect for improving services in Bangladesh, India, Nigeria and Oman [18,26,27,36,37,49]. In a study in Bangladesh, clients considered reducing waiting time more important than increasing consultation time [18]. The provision of personalized care and constant attention were identified as probable causes that led to women being significantly more satisfied with care in lower level centers as compared to higher level hospitals in Sri Lanka [30]. Promptness also included lack of prompt referral, which was a cause for dissatisfaction with services in one study in Nigeria [50].

#### Interpersonal behavior

Evidence on interpersonal aspects of care as determinants of maternal satisfaction was generated in 22 studies from 18 countries. Being treated with dignity, respect and courtesy was a key determinant of maternal satisfaction [17,19,20,22,26,32,33,36,42,44,45,47,50-52]. Therapeutic communication (listening, politeness, prompt pain relief, kindness, approachability and smiling demeanor), caring behavior (attentive to needs, making clients feel accepted and coaxing clients) and interpersonal skills of staff (staff confidence and competence) were significant themes that were identified as influencing client’s satisfaction with care in Ghana, Lebanon and Gambia [23,24,34]. This aspect appeared much more important to patients than technical competence of providers in Bangladesh [18]. The use of praising words by the medical staff or by the obstetrician or midwife during delivery encouraged women and boosted their self-esteem, as reported in a study in Lebanon [24]. In fact women chose to repeat the same provider for their next delivery if s/he was comforting and encouraging to them [24]. On the other hand, staff unfriendliness, negative attitude and impatience was a major cause for dissatisfaction with services and avoidance of use in Nigeria, Zambia, Pakistan, Ghana and Turkey [17,23,25,43,49,53,54].

#### Privacy

Privacy is a key requirement of women utilizing maternal care services, for physical examinations as well as the delivery process itself. A sense of shame is also attached to the process of physical examination and also procedures like perineal shaving, thereby increasing women’s discomfort and diminishing their satisfaction levels [24]. Inadequate privacy during antenatal checkup and counselling was associated with women’s poor perception of services [34,55]. Maintenance of privacy via a separate room or screen for examination or delivery was a significant determinant of satisfaction with maternal health services in Bangladesh and India [18,36,37]. Lack of confidentiality during checkups and deliveries, on the other hand, caused dissatisfaction with services in Nigeria and Cuba [44,50].

#### Perception of ‘good’ care or provider competency

Women were more satisfied with maternal health services when they perceived the technical quality of care to be ‘good’ or the provider to be technically competent. Completeness of procedures, good medicine and advice were perceived as ‘good care’ in India [36]. Lack of congruence between care expected and care actually received also determined women’s level of satisfaction, as found in a study in Ghana [23]. Length of consultation was a significant predictor of maternal satisfaction in studies in Bangladesh, China, Vietnam, Nigeria, India, Iran and Gambia [18,20,28,32,34,41,42,50].

Perceived neglect in care, including delay in attending to the client and not involving the client in care, poor handling during labor and mistakes in test results also adversely affected satisfaction with services in Ghana and Nigeria [23,25,33,50]. Overcrowding and unnecessarily prolonged facility stays also reduced maternal satisfaction in Ghana and Malawi [19,33].

Perceived competence was associated with provider qualification or previous experience, and was a significant factor in maternal satisfaction in Vietnam, Cuba, Thailand, Nigeria, Kenya, India and China [20,32,36,41,44,52]. Maternal satisfaction increased significantly in Iran when a new model of delivery care, based on women’s perceived care need, was introduced at a tertiary facility [56]. This indicates the positive effect of responsive care on maternal satisfaction.

#### Cognitive support

Provision of cognitive support through effective communication and sharing adequate information with women about their condition or the care required, emerged as a critical determinant of satisfaction with maternal care, as seen in studies in Ghana, Malawi, Nigeria and Iran [19,28,32,40,51]. Counseling by the provider, the process of imparting information, consultation in decisions regarding care, and transparent mechanisms for registering patient feedback were all important aspects of cognitive support [19,23,26,29,30,32,41,44,46,47,50-52,57]. One of the key reasons for satisfaction with group prenatal care in a trial in Iran was the information provided on care during this period [28]. In a study in Oman, women’s satisfaction related as much to the content of messages as to the process of imparting it, such as the provider’s commitment, availability of time and overcoming any language barrier [26]. In Ghana, clients who had information during labor felt involved in their care and this contributed to their satisfaction with care [23].

#### Emotional support

Support provided by a companion of the woman's choice during labor and delivery has a significant positive effect on her satisfaction with the overall birth experience, as found in studies in Brazil and Malawi [19,58]. In a study in Zambia one of the major complaints with services was ‘being left alone in labor too long’ [17]. Positive benefits of birth companionship was evidenced in terms of shorter labor, lesser need for pain relief and greater birth satisfaction among women with birth companions during labor in studies in Mexico, Jordan and United Arab Emirates [59-61]. In a non-randomized comparison study in Jordan it was found that women who had support from a female relative during labor were less likely to use pharmacological pain relief and more likely to report a good birth experience [60].

#### Preference for female providers

Preference for female providers emerged as a significant determinant of satisfaction with care in developing country contexts, as evidenced in studies in Nigeria, Lebanon, Senegal, India, Saudi Arabia and Thailand [24,36,43,44,47,62]. A study in India found higher preference for female doctors on account of greater comfort felt by women in communicating with them, greater sense of privacy and the perception that lady doctors are more patient, ‘deliver properly’ and are good for examinations [36]. Women in Saudi Arabia and Thailand also felt that female providers have greater understanding of the physical and psychological needs of pregnant women [44]. This could, however, be overridden by concern for safe medically-attended deliveries in hospitals [24,44].

### Outcome

#### Delivery outcome

Maternal and newborn outcomes in terms of survival and health of mothers and newborns (for example, mother alive in spite of fetal loss; baby alive and healthy) affected satisfaction with care in Gambia, Ghana, India and Thailand [22,33,36,39].

### Other factors

#### Convenience of access

Convenience of access to maternity care is an important determinant of maternal satisfaction in developing countries, as reported by a number of studies [33,39,63,64]. Access included both distance and connectivity (availability of public transport between residence and facility). In a study on patient perception of antenatal care quality in selected private facilities in Nigeria, location of the facility near the residence and convenient timings led to greater satisfaction among women utilizing it for antenatal services [50]. One of the major reasons for satisfaction with home delivery by traditional birth attendants in Pakistan was the convenience of access as they lived in the neighborhood [53]. Not all evidence has been conclusive on convenience of access significantly influencing maternal satisfaction, as studies in Bangladesh and Sri Lanka found that it did not have any significant effect on maternal satisfaction [27,30,41].

#### Cost of care

Significant associations between cost and maternal satisfaction and the utilization of care in both home and institutional births were found in studies in Nigeria, Zambia, Kenya, Egypt, India, Pakistan, Gambia and Ghana [17,22,33,36,48,52,53,63,64]. Affordable care was a significant determinant of satisfaction with maternal care services in both facility and home deliveries in India, Kenya and Pakistan [36,52,53].

Besides overall cost of care, affordable drugs, availability of finance for healthcare and transparency in financial transactions also influenced satisfaction with care in Nigeria, Gambia, Thailand, Vietnam, Iran, China, Ghana and Argentina [20-23,39,44,46,50]. Availability of free medicines in the facility significantly enhanced maternal satisfaction with care in Gambia [22].

#### Maternal characteristics

Maternal characteristics also affected women’s perceived satisfaction with care. Maternal age and education was positively associated with maternal satisfaction, possibly because of greater experience and maturity [34,42,65]. Studies in Nigeria and Sri Lanka found multiparous women were more satisfied with care as compared to primiparae women [30,32]. Maternal satisfaction in Kenya was also significantly determined by whether the pregnancy was intended or not [52]. Women’s level of stress during delivery and in the postpartum period also significantly influenced satisfaction with care in Thailand, Taiwan and Nigeria [21,39,65].

#### Socio-economic and cultural determinants

Among socio-economic and cultural factors, ethnicity influenced maternal satisfaction in Kenya and Sri Lanka, while religion emerged significant in a study in Nigeria [30,32,52]. Women’s education levels negatively affected their satisfaction with maternal care as per studies in India, Nigeria, Tanzania, Ghana and Zambia [17,23,32,37,66]. Mother’s expectation of baby’s gender affected satisfaction with services in studies in Thailand and Saudi Arabia [39,47]. Positive impact of the first experience of care influenced perceived satisfaction with care, as diminishing satisfaction was found with increasing familiarity in a facility-based study in Nigeria [32].

## Discussion

This review is one of the first attempts to capture the wide spectrum of determinants of maternal satisfaction in developing countries. Commonalities in findings across studies in different countries, with similar research questions and socio-economic contexts lends credence to the synthesis in representing the broader context of developing countries.

Satisfaction ratings by women are high across most studies – this could be because of lack of awareness and exposure in largely low literacy contexts of developing countries. However, further analysis of maternal satisfaction ratings is required to substantiate this observation, which was beyond the scope of our review. In India, the scheme of monetary incentive for institutional delivery has accelerated utilization of facilities for childbirth, without necessarily improving quality of care [67]. On the contrary, service quality is strained on account of overcrowding, especially in referral facilities [67].

To analyze the relative significance of the determinants, we categorized those reported in five or more studies as major, while others were categorized as minor (Table 2). Determinants reported by a relatively larger number of studies include interpersonal behavior, waiting time before admission or consultation and perceived provider competency.

**Table 2:** **Major determinants of maternal satisfaction identified in the review**

**Quality of Care Framework**	**Themes**	**Major determinants**	**Minor determinants**
		**(reported in five or more studies)**	**(reported in fewer than five studies)**
**Access**	**Convenience of access**	Distance & transport connectivity	Access to drugs; opening and closing timings
**Structure**	**Physical environment**	-	Good infrastructure, electricity, water supply, waiting area, seating arrangement, facility management (patient access and consultation systems, information channels, financial management)
	**Cleanliness**	Cleanliness, clean toilets, hygiene maintenance	Housekeeping services
	**Human resources**	-	Staffing adequacy, availability of doctors to manage emergencies, availability of nursing personnel
	**Medicines, supplies & services**	Availability of drugs and equipment	Availability of - ‘good’ services; ambulance services; lab services; blood supply & transfusion services
**Process of care**	**Promptness of care**	Waiting time before admission or consultation	Timely attendance, constant attention; prompt referral; immediate contact with newborn
	**Interpersonal behavior**	Respectful behavior by doctors, nurses and support staff	Therapeutic communication; encouragement during delivery
	**Privacy & confidentiality**	Privacy & confidentiality during examinations and delivery	-
	**Perception of ‘good’ care**	Length of consultation; completeness of procedures; perception of negligent care; perceived provider competence	-
	**Cognitive support**	Prenatal counseling and health education	Information shared with women about their condition and care; sense of ‘participation’ in the process; culturally sensitive communication
	**Emotional support**	Birth companion of choice	Social networks of expectant mothers; support from family members
	**Preference for female providers**	Preference for female providers	-
**Cost**	**Cost of care**	Financial cost of care	Affordable drugs, availability of financial support for care
**Outcome**	**Delivery outcome**	-	Healthy newborn; survival of maternal illness; successful labour outcome for mother & baby
**Other determinants**	**Maternal characteristics**	-	Age, parity, whether pregnancy was intended, stress during delivery and postpartum
	**Socio-cultural determinants**	Literacy	Ethnicity, religion, type of locality where facility is located, expectation of baby’s gender, positive impact of first experience of care

The most striking finding was interpersonal behavior as the most widely reported determinant of satisfaction. The largest body of evidence generated in the review revolves around provider behavior in terms of courtesy and non-abuse. It shows the importance women attach to being treated with courtesy and empathy, irrespective of socio-cultural or economic context. Women identify ‘being treated as a human being’ as one of the benchmarks of high quality care [68]. Across the world, women seek dignity and respect while undergoing maternity care. Provider behavior and attitudes are therefore major determinants of utilization of skilled maternity care [18,69]. Increasing documentation of neglect and intentional abuse and humiliation of women during childbirth in countries across the world indicates that this is indeed a major factor inhibiting uptake of services [70,71]. In this context it is important that efforts to improve access and availability of skilled delivery care to women in developing countries focus equally on sensitizing providers on respectful care.

Labor and childbirth is a particularly vulnerable time for women and the need for attention and care is very important [19]. It is but natural that women attach great value to the care they receive during this time. Their satisfaction hinges upon timely and ‘good’ quality care, as per the woman’s expectations. The perception of ‘good’ care is therefore a significant determinant of maternal satisfaction, with four sub-themes emerging as major determinants – length of consultation, completeness of procedures, perception of negligent care (which diminishes satisfaction) and perceived provider competence. Maintenance of privacy and confidentiality was also a marker of good care and was another important determinant of satisfaction. Provider’s respect for privacy and confidentiality emerged as a statistically significant predictor of maternal satisfaction [18].

Cognitive and emotional support plays a crucial role in influencing women’s satisfaction with care during pregnancy and childbirth. Information and advice, along with emotional support, comfort measures and communication may reduce anxiety and fear and associated adverse effects during labor [72]. Prenatal counseling is a major determinant of satisfaction, as it is critical for a woman’s understanding of her health condition and her participation in the pregnancy and delivery process [26,29]. Similarly emotional support is also essential for reassurance and comfort of birthing women. The World Health Organization has recommended that the parturient woman should be accompanied by people whom she trusts and feels safe with, such as family members [59,73]. There is significant evidence from developing countries around shorter labor and lesser need for pain relief associated with psychosocial support by a birth companion [59-61]. Preference for female provider for maternity care could be a culturally influenced determinant of maternal satisfaction as it would decrease the sense of embarrassment and fear which parturient women may feel in a facility [73].

The major structural determinant of maternal satisfaction emerging from the review is ‘the availability of drugs and equipment’. A possible reason why other structural elements did not emerge as major determinants could be that women from poorer communities in developing countries have limited access to public services and almost no sense of entitlement to healthcare [74]. Even basic facilities available would be satisfactory to them. This is substantiated with the finding that women in their first visit to the facility express greater satisfaction with services owing to the positive impact of first experience of care, as compared to those making repeat visits [31]. But for those who have accessed care multiple times, poor availability of drugs or equipment would be a serious quality gap, as has emerged in the evidence from literature.

Among other determinants, cleanliness is another important structural determinant of maternal satisfaction [23,75]. Evidence around outcome of care as a determinant of maternal satisfaction was comparatively lesser. Access to care and cost of care have emerged as determinants of maternal satisfaction. In low resource contexts of developing countries, availability and access to medical care are significant issues, along with affordability of care, especially for the poor.

A majority of the studies investigated association between maternal satisfaction and socio-demographic characteristics of the women. Literacy emerged as a major factor influencing satisfaction with maternity care. Research has shown that the duration of women’s schooling changes their perceptions about health services, leading to better knowledge and better utilization of most types of health care [76].

### Policy and program implication

The review findings have revealed that women’s experience of care is affected by a wide range of determinants. This could influence their future utilization of care. Maternal health programmes and policies in developing countries therefore must take into account women’s perspective on the care they need and their feedback on the services they receive. It is important for any such programs to have a robust system of obtaining client feedback and utilizing such information to improve services. Aspects of interpersonal care that have emerged as determinants of satisfaction with care in a large number of studies need to be paid special attention. International research programs have developed toolkits on ‘caring behaviors’ that can be utilized for building the capacity of local maternity care providers in developing countries to sensitize them on improving interpersonal behavior and communication skills [77]. Providing culturally sensitive care, such as by female providers, is also important to improve institutional deliveries in some developing countries where the provider’s gender has emerged as an important determinant of maternal satisfaction.

### Limitations of the review

Though we searched multiple databases, there is a possibility that we may have missed out some studies, as our search criteria were broad, in keeping with the review objective. We did not conduct any meta-analysis or combining of results from studies of varying quality using weights or scores; this has resulted in lack of any quantifiable results on strength of associations of determinants with maternal satisfaction. Another limitation was the review’s inclusive approach to study selection, incorporating various methodologies and not considering quality assessment as a screening criterion. The only purpose was to achieve a wide range of determinants, regardless of the quality of studies.

The reviews aims to represent developing country contexts, but inter- and intra-country socio-cultural variations are bound to play a differentiating role and may affect the generalizability of the results. Most studies explored satisfaction in facility settings or facility-based care as opposed to home deliveries or home-based care. Hardly any studies captured information on satisfaction in the context of home deliveries, which remain significantly high in developing countries. Meta-analysis of satisfaction scores was inhibited by the inclusion of studies of diverse design and quality. Qualitative studies investigated issues related to women’s satisfaction in much greater detail, but could not provide any objective assessment of level of satisfaction. Mixed methods studies were at an advantage in this regard, as they were able to substantiate women’s ratings of satisfaction with in-depth analysis of their experience of care.

## Conclusions

The growing demand for health care coupled with constrained resources, and evidence of variations in maternity care practices have increased governments’ interest in measuring and improving quality of institutional delivery care services in many countries in the developing world [78]. Incorporating patients’ views into quality assessments is critical in making health services more responsive to people’s needs [79]. This review is useful in indicating the aspects of care that need to be focused on while assessing the quality of care or taking action to improve it.

There is need for more research into maternal satisfaction in developing countries, where safe deliveries remain a major problem and barriers to utilization of institutional deliveries pose a major challenge for healthcare programs. Further research into maternal satisfaction could be made more policy-relevant by assessing the relative strength of various determinants in influencing maternal satisfaction; this could help in prioritizing appropriate corrective interventions for improved quality of care.

## Additional file

Additional file 1: Formats for data extraction. Additional file 2: Table S2. Summary of studies included in the review.
