# Supplementary material for: Determinants of women’s satisfaction with maternal health care: a review of literature from developing countries
Source: BMC Pregnancy Childbirth. 2015 Apr 18;15:97. doi: 10.1186/s12884-015-0525-0 (PMC4417271; doi:10.1186/s12884-015-0525-0)
Supplement: Additional file 1: — Formats for data extraction. [file 12884_2015_525_MOESM1_ESM.pdf]

Formats for data extraction

1. Study Summary Listing Format

Study ID	Year	Publication type	About Study	Maternal satisfaction/perception measured through	Inclusion decision
First Author & Title			*Study type, objective & setting*	*Details of methodology / tool*	*Decision:*<br>*Reason:*

2. Detailed Data extraction format

Study / design	Participants	Analysis / Interventions	Outcome measures
Study ID/ year<br><br>Country:<br><br>Region:<br><br>Time frame:<br><br>Location / setting:<br><br>Focus / primary aim of study:<br><br>Determinants of satisfaction:<br><br>Study design:<br><br>Funding:<br><br>Quality: *high / moderate / low from assessment below*	<u>Population</u>: *number and description(age, gender, ethnicity, socio-economic characteristics)*<br><br>Selection criteria:<br><br><u>Health system information:</u><br><br>Health professionals / services involved:<br><br>Subgroups (if any):	Data collection methods:<br><br>Instrument for assessing patient satisfaction/ feedback:<br><br>Data analysis methods:<br><br>Details of data quality management:	Primary outcomes:<br><br>Outcomes relating to determinants of maternal satisfaction:<br><br>Other outcomes:
